# Identifying critical windows for macrosomia prevention: a study on the association between phase-specific dynamic BMI changes and macrosomia risk

**DOI:** 10.3389/fendo.2026.1875915

**Published:** 2026-08-11

**Authors:** Huiling Qu, Yanna Zhou

**Affiliations:** Department of Obstetrics and Gynecology, Jinshan Branch of Shanghai Sixth People’s Hospital, Shanghai, China

**Keywords:** BMI increment, gestational BMI, macrosomia, pregnancy outcomes, pre-pregnancy body mass index (BMI), weight management

## Abstract

**Objective:**

To examine the correlation among pre-pregnancy body mass index (BMI), BMI increments during different gestational periods, and the occurrence of macrosomia in singleton full-term pregnant women without complications. Additionally, we aimed to assess the predictive performance of these BMI-related indicators for macrosomia, thereby identifying key time windows for targeted gestational weight management.

**Methods:**

An analytical approach was adopted, entailing a retrospective evaluation of clinical data from 4,218 pregnant women without complications who underwent regular prenatal examinations and singleton full-term delivery during the period between January 2019 and December 2025 at the Jinshan Branch of Shanghai Sixth People’s Hospital. Based on neonatal birth weight, a case-control design was employed, selecting 200 cases of macrosomia (macrosomia group) and 250 cases of normal birth weight infants (control group). Data including pre-pregnancy BMI, baseline BMI at the first prenatal visit (10–13 weeks), and BMI at various stages of mid- and late-pregnancy (20, 24, 28, 32, 36 weeks, and before delivery) were collected to calculate BMI increments for each stage. The predictive efficacy of individual BMI indicators and a combined model for macrosomia was evaluated using independent sample t-tests, Pearson correlation analysis (PCA), and receiver operating characteristic (ROC) curves.

**Results:**

(1) Increased pre-pregnancy BMI was significantly and positively correlated with an increased macrosomia risk. Pre-pregnancy overweight (OR = 2.63) and obesity (OR = 9.06) emerged as independent risk contributors for macrosomia (P < 0.01). (2) Baseline BMI at 10–13 weeks, along with BMI increments during the 32–36 weeks period and the period from 36 weeks to delivery, exhibited a significant increase in the macrosomia group in comparison to the control group (P < 0.05). (3) The analysis of correlation demonstrated a moderate positive correlation between baseline BMI at 10–13 weeks and neonatal birth weight (r = 0.386, P < 0.001). However, no significant correlation was identified between mid-pregnancy BMI increments and birth weight (P > 0.05). (4) Utilising ROC curve analysis, it was ascertained that baseline BMI at 10–13 weeks exhibited the most optimal predictive efficacy among the individual indicators (AUC = 0.73), with the late-pregnancy increment indicators following in succession. The combined predictive model, integrating early-pregnancy BMI and late-pregnancy BMI increments, achieved a superior AUC of 0.84.

**Conclusion:**

Pre-pregnancy BMI, early-pregnancy (10–13 weeks) BMI, and late-pregnancy (32 weeks to delivery) BMI increments are critical predictors of macrosomia in pregnant women without complications. Specifically, baseline BMI at 10–13 weeks serves as a core indicator for early risk identification. Clinical interventions should focus on these two critical stages—early and late pregnancy—to implement individualized weight management, thereby effectively reducing the incidence of macrosomia and improving maternal and neonatal outcomes.

## 1 Introduction

Fetal macrosomia is a common adverse obstetric outcome that significantly increases the risk of maternal complications, including shoulder dystocia, severe birth canal lacerations, uterine atony, and postpartum hemorrhage, as well as neonatal birth injuries such as intracranial hemorrhage, clavicular fracture, and brachial plexus injury, thereby posing serious threats to short-term maternal and neonatal health (1). Consequently, early prediction and precise prevention of macrosomia have become key priorities in perinatal care. With global lifestyle changes, the prevalence of being overweight and obesity in the pre-pregnancy population has been steadily rising (2). Body mass index (BMI), a pivotal metric of maternal nutritional status, exhibits a robust correlation with fetal intrauterine growth and development (3). Dynamic changes in maternal weight during pregnancy influence the risk of pregnancy-related maternal complications and fetal growth trajectories (4). Existing evidence shows that being overweight or obese before pregnancy significantly increases the risk of the offspring having a low birth weight, and that gaining too much weight during pregnancy further increases this risk (5). Therefore, prenatal prediction of macrosomia holds important clinical significance (6). Although previous studies have established gestational weight gain in the pre-pregnancy overweight and obese categories as a major risk factor for macrosomia, most have primarily focused on total weight gain, with relatively limited attention paid to BMI increments across different gestational stages (7, 8). Moreover, the lack of clearly defined key intervention time windows has restricted the development of individualized clinical management strategies (9, 10). This research study has been conducted with the objective of systematically investigating the associations of pre-pregnancy BMI, early-pregnancy BMI (10–13 weeks), and BMI increments at various gestational stages with the risk of macrosomia in uncomplicated singleton full-term pregnancies. In addition, the predictive performance of these BMI-related indicators will be evaluated. The goal is to identify critical time points for gestational weight management, with a view to providing evidence-based support to facilitate early screening, targeted interventions, and improved outcomes for both mother and infant.

## 2 Materials and methods

### 2.1 Study population

This study retrospectively enrolled pregnant women without pregnancy-related complications who underwent regular prenatal examinations and delivered singleton term infants during the period between January 2019 and December 2025 in the Jinshan Branch of Shanghai Sixth People’s Hospital.

Inclusion criteria were defined as such: (1) singleton full-term pregnancy (37–42 weeks of gestation) with complete antenatal management and delivery at the study center; (2) initial prenatal assessment performed at 10–13 weeks of gestation, with comprehensive follow-up data at 20, 24, 28, 32, and 36 weeks, as well as prior to delivery; (3) no history of pre-existing disorders or gestational complications; (4) absence of fetal structural abnormalities or chromosomal defects; and (5) live birth with available and complete neonatal birth weight information.

Exclusion criteria were set as such: (1) multiple pregnancy, preterm birth (<37 weeks), or post-term pregnancy (>42 weeks); (2) presence of pregnancy-related diseases, complications, or use of special medications during pregnancy; (3) incomplete prenatal data, missing weight measurements, or inaccurate gestational age estimation; (4) fetal structural abnormalities or other anomalies; and (5) inability to determine neonatal birth weight.

**Figure 1:**
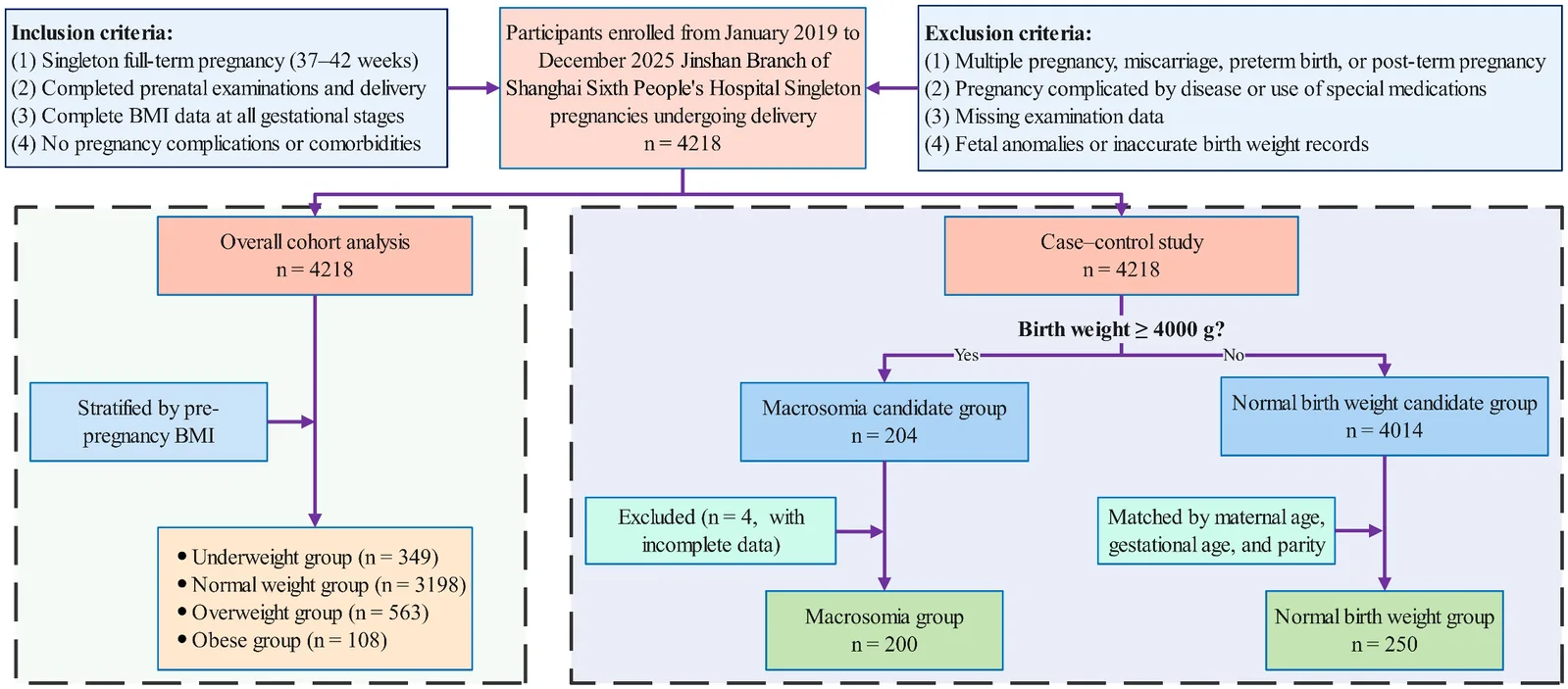
Workflow of participant selection and study design.

After screening, 4,218 eligible cases were identified, among which 204 cases of macrosomia were recorded (incidence rate: 4.84%). After excluding 4 cases with incomplete data, 200 cases were finally included in the macrosomia group. To ensure comparability between cases and controls and minimize potential selection bias, this study adopted a restricted random sampling strategy for control selection. Specifically, from the 4,014 pregnant women who delivered neonates with normal birth weight, 250 were randomly selected as the control group, matched according to the same criteria as the case group (including age, gestational age, gravidity, and parity). To facilitate comprehension, the overall research workflow has been illustrated in **Figure 1**.

### 2.2 Determination of gestational age

Gestational age was established and subsequently adjusted using crown–rump length (CRL) measurements obtained via B-mode ultrasonography during early pregnancy (6–13 weeks of gestation), to ensure the reliability and precision of gestational age assessment.

### 2.3 Study methods

#### 2.3.1 Clinical data retrieval

Clinical records were retrieved from two sources: the hospital’s electronic medical record database and the Shanghai Maternal and Child Health Information System. The baseline characteristics encompassed maternal age, obstetric history, height, pre-pregnancy weight, weight at the first prenatal visit, and body weight recorded at each gestational stage. A comprehensive documentation of perinatal outcomes was conducted, encompassing neonatal birth weight, mode of delivery, and gestational age at delivery, in the immediate postpartum period. The accuracy and thoroughness of the data were ensured by the independent extraction and cross-checking of the data by two trained obstetricians. In instances of discrepancy, the final data were reviewed and confirmed by a senior obstetrician.

#### 2.3.2 BMI calculation and definition of BMI increment

The following formula was used to calculate the BMI: BMI = body weight (kg)/height² (m²). BMI values were obtained for each participant at the following gestational stages: pre-pregnancy, the initial prenatal visit (10–13 weeks of gestation), and at 20, 24, 28, 32, and 36 weeks, as well as immediately prior to delivery. BMI increment was defined as the change in BMI between two successive prenatal assessments. Specifically, BMI increments were derived across six gestational periods: 13–20 weeks, 20–24 weeks, 24–28 weeks, 28–32 weeks, 32–36 weeks, and from 36 weeks until delivery. All weight measurements were performed using a calibrated, standardized scale. Participants were required to remove heavy garments and fast prior to measurement. The height of the participants was measured using a fixed stadiometer, with a measurement precision of 0.1 cm. Body weight was recorded to the nearest 0.1 kg. All BMI calculations and increment determinations were conducted using standardized analytical software by trained staff to reduce potential measurement bias.

#### 2.3.3 Neonatal birth weight classification

‘Macrosomia’ was defined as a neonatal birth weight of at least 4,000 g, whereas ‘normal birth weight’ as a measurement ranging from 2,500 to 4,000 g. The assessment of neonatal birth weight was conducted immediately after delivery by trained midwives utilizing a standardized scale.

### 2.4 Statistical analysis

All the statistical analyses were conducted with the use of SPSS software (version 27.0; IBM Corp., Armonk, NY, USA). Continuous variables were expressed as the mean ± standard deviation (mean ± SD). A subsequent analysis involved the comparison of baseline BMI and BMI increments between the macrosomia and control groups, utilizing independent-samples t-tests. Pearson correlation analysis was applied to assess the relationships between BMI at the first prenatal visit (10–13 weeks of gestation) and stage-specific BMI increments with neonatal birth weight. The strength of the correlations was then quantified using the correlation coefficient (r), with r > 0.3 and P < 0.05 considered indicative of clinical relevance. The ability of BMI-related variables to predict macrosomia was assessed using receiver operating characteristic (ROC) curve analysis. The area under the curve (AUC) and its 95% confidence interval (CI) were then calculated. A two-tailed P value of less than 0.05 was considered statistically significant.

## 3 Results

### 3.1 General characteristics

The present study comprised a total of 4,218 pregnant women. The classification of participants was determined by their pre-pregnancy BMI values, which were then used to determine their categorization according to the 2021 guidelines of the Chinese Nutrition Society. The categorization system employed in this study is as follows: obese (BMI ≥ 28.0 kg/m²), overweight (24.0 ≤ BMI < 28.0 kg/m²), normal weight (18.5 ≤ BMI < 24.0 kg/m²), and underweight (BMI < 18.5 kg/m²). The distribution of participants across these categories was as follows: The underweight group comprised 349 subjects (8.27%), the normal-weight group 3,198 subjects (75.83%), the overweight group 563 subjects (13.35%), and the obese group 108 subjects (2.55%). The preliminary characteristics of the entire study cohort are outlined in **Table 1**.

**Table 1:** Baseline characteristics of participants according to pre-pregnancy BMI categories.

Group (pre-pregnancy BMI category)	N (%)	Age (years, median [IQR])	Pre-pregnancy BMI (kg/m², mean ± SD)	Gravidity (mean ± SD)	Parity (mean ± SD)	Education (≤ junior college, %)
Underweight (<18.5)	349 (8.27%)	27 (20–33)	17.8 ± 2.1	2.58 ± 0.60	1.30 ± 0.49	20.34%
Normal weight (18.5–24.0)	3198 (75.83%)	28 (22–35)	21.7 ± 2.2	2.37 ± 0.48	1.34 ± 0.49	25.89%
Overweight (24.0–28.0)	563 (13.35%)	31 (26–36)	25.5 ± 1.6	2.90 ± 0.94	1.93 ± 0.36	38.19%
Obese (≥28.0)	108 (2.55%)	33 (28–38)	29.4 ± 3.3	3.04 ± 0.87	2.03 ± 0.29	39.81%

### 3.2 Incidence of macrosomia across different pre-pregnancy BMI categories

Analyses revealed incidence rates of macrosomia to be 1.43%, 3.72%, 9.24%, and 25.93% in the underweight, normal-weight, overweight, and obese groups, respectively. Furthermore, as BMI increases, the incidence rate of macrosomia shows a significant upward trend (see **Table 2**). Using the normal-weight group as the reference (odds ratio [OR] = 1.00), the risk of macrosomia was significantly lower in the underweight group (OR = 0.38, 95% CI: 0.15–0.93, P < 0.05). In contrast, the risk was significantly higher in the overweight group (OR = 2.63, 95% CI: 1.88–3.70, P < 0.001) and further increased in the obese subjects (OR = 9.06, 95% CI: 5.67–14.46, P < 0.001). The findings indicate a strong positive correlation among subjects with a BMI above the median value at the time of conception and the risk of macrosomia, thereby demonstrating a clear dose-response relationship.

**Table 2:** Incidence of fetal macrosomia according to pre-pregnancy BMI categories.

Group (pre-pregnancy BMI category)	Total (n)	Macrosomia (n)	Incidence (%)	Crude OR	95% CI
Underweight (<18.5)	349	5	1.43	0.38	0.15–0.93
Normal weight (18.5–24.0)	3198	119	3.72	1.00	Reference
Overweight (24.0–28.0)	563	52	9.24	2.63	1.88–3.70
Obese (≥28.0)	108	28	25.93	9.06	5.67–14.46

### 3.3 Differences in BMI at first prenatal visit and stage-specific BMI changes between groups

The baseline profiles of participants in two distinct groups were analysed and compared: the macrosomia group and the normal birth weight group. The investigation revealed no statistically significant differences between the two groups with respect to maternal age, gestational age, gravidity, parity, or educational level (all P > 0.05). However, a marked elevation in pre-pregnancy BMI was observed in the macrosomia group compared with the normal birth weight group (24.60 ± 2.37 vs. 22.65 ± 2.66, t = 8.21, P < 0.001), suggesting an elevated baseline BMI among women delivering macrosomic neonates (see **Table 3**).

**Table 3:** Comparison of baseline characteristics between the macrosomia group and the normal birth weight group.

Variable	Macrosomia group (n = 200)	Normal birth weight group (n = 250)	t/χ² value	P value
Pre-pregnancy BMI (kg/m², mean ± SD)	24.60 ± 2.37	22.65 ± 2.66	8.21	<0.001
Age (years, mean ± SD)	28.6 ± 3.3	28.1 ± 3.7	1.51	>0.05
Gestational age (weeks, mean ± SD)	39.1 ± 1.1	38.9 ± 1.3	1.77	>0.05
Gravidity (mean ± SD)	2.43 ± 0.41	2.41 ± 0.27	0.59	>0.05
Parity (mean ± SD)	1.31 ± 0.81	1.25 ± 0.67	0.84	>0.05
Education (≤ junior college, %)	23.50%	25.20%	0.17	>0.05

Further evaluation of BMI trajectories across gestation indicated that BMI levels in the macrosomia group continued to be significantly elevated at the first prenatal visit (10–13 weeks of gestation) in comparison with those in the normal birth weight group (P < 0.001).

Conversely, there were no statistically significant differences in BMI increments observed between groups during mid-pregnancy, including the intervals of 13–20 weeks, 20–24 weeks, and 24–28 weeks (all P > 0.05). However, in late gestation, BMI increments in the macrosomia group were significantly greater than those observed in the normal birth weight group during both the 32–36 weeks interval and the period from 36 weeks until delivery (both P < 0.01). These findings indicate that the development of macrosomia is associated not only with elevated pre-pregnancy and early-pregnancy BMI, but also with excessive BMI gain during late gestation. This underscores the critical role of weight control in late pregnancy for macrosomia prevention (see **Table 4**).

**Table 4:** Baseline BMI and stage-specific BMI increments during pregnancy in the macrosomia and normal birth weight groups.

BMI variables (kg/m², mean ± SD)	Macrosomia group	Normal birth weight group	P value
BMI at 10–13 weeks	24.89 ± 2.37	22.74 ± 2.66	<0.001
BMI increment (13–20 weeks)	1.13 ± 0.79	1.09 ± 0.87	>0.05
BMI increment (20–24 weeks)	1.02 ± 1.15	0.85 ± 1.11	>0.05
BMI increment (24–28 weeks)	0.53 ± 0.60	0.52 ± 0.51	>0.05
BMI increment (28–32 weeks)	0.66 ± 0.84	0.51 ± 0.88	>0.05
BMI increment (32–36 weeks)	0.43 ± 0.55	0.28 ± 0.52	<0.01
BMI increment (36 weeks to delivery)	0.44 ± 0.34	0.29 ± 0.35	<0.001

### 3.4 Correlation between BMI at first prenatal visit, stage-specific BMI increments, and neonatal birth weight

Following a thorough adjustment process for potential confounders, including maternal age, gestational age, gravidity, and parity, correlation analyses were conducted to investigate the relationships between BMI at the first prenatal visit (10–13 weeks of gestation), stage-specific BMI increments, and neonatal birth weight (see **Table 5**). The findings suggested that BMI at the initial antenatal visit (10–13 weeks) demonstrated a moderate positive correlation with neonatal birth weight (r = 0.386, R² = 0.149, 95% CI: 0.304–0.462, P < 0.001), indicating that BMI in the early stages of pregnancy may possess predictive capabilities concerning neonatal birth weight.

**Table 5:** Correlation between BMI at the first prenatal visit, stage-specific BMI increments, and neonatal birth weight.

Variable	Pearson correlation coefficient (r)	Coefficient of determination (R²)	95% CI	t value	P value
BMI at 10–13 weeks	0.386	0.149	0.304–0.462	8.853	<0.001
BMI increment (13–20 weeks)	0.047	0.002	−0.045–0.139	1.002	0.317
BMI increment (20–24 weeks)	0.059	0.004	−0.033–0.151	1.255	0.210
BMI increment (24–28 weeks)	0.019	0.000	−0.074–0.111	0.402	0.688
BMI increment (28–32 weeks)	0.067	0.004	−0.026–0.158	1.413	0.158
BMI increment (32–36 weeks)	0.171	0.029	0.080–0.259	3.667	<0.001
BMI increment (36 weeks to delivery)	0.200	0.040	0.109–0.287	4.318	<0.001

(1) Criteria for interpreting correlations: A correlation was considered clinically relevant when r > 0.3 and P < 0.05, indicating a statistically significant positive relationship. When r < 0.3 but P < 0.05, the correlation was regarded as statistically significant but lacking clinical relevance. Conversely, when P > 0.05, the null hypothesis was rejected, indicating a statistically significant association. (2) Coefficient of determination: The coefficient of determination (R²) denotes the squared value of the Pearson correlation coefficient (r) and reflects the proportion of variance in neonatal birth weight explained by each BMI-related variable. A higher R²indicates greater explanatory capacity. (3) Statistical parameters: The t values and 95% CI were derived using the Pearson correlation method, with degrees of freedom defined as df = n − 2 = 448. (4) Definition of BMI-related variables: BMI was determined by dividing the body weight (kg) by the square of the height (m²). BMI increment was delineated as the change in BMI between two successive prenatal assessments.

Further examination of BMI increments across gestational stages demonstrated that BMI increments during mid-pregnancy, including 13–20 weeks, 20–24 weeks, 24–28 weeks, and 28–32 weeks of gestation, exhibited no significant association with neonatal birth weight (all P > 0.05). In contrast, during late pregnancy, BMI increment at 32–36 weeks was weakly but significantly positively correlated with neonatal birth weight (r = 0.171, P < 0.001), and this relationship became more pronounced during the period from 36 weeks to delivery (r = 0.200, P < 0.001).

These findings demonstrate a greater influence of maternal BMI in early pregnancy, in conjunction with BMI gain during late gestation, on neonatal birth weight, while BMI changes in mid-pregnancy appear to have a comparatively minor effect.

**Figure 2:**
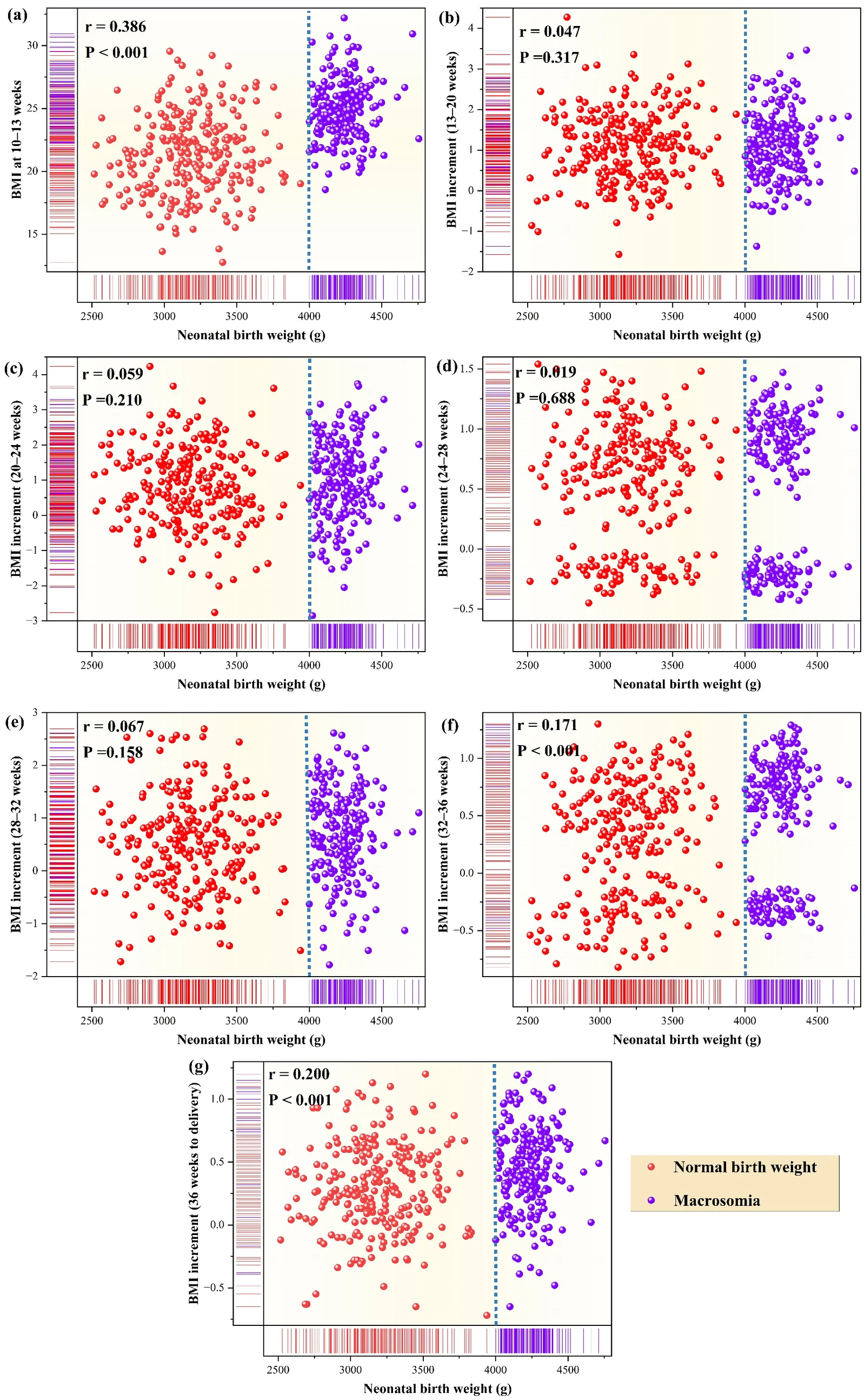
Correlations between BMI at the first prenatal visit (10–13 weeks of gestation), stage-specific BMI increments, and neonatal birth weight. Panels **(a–g)** correspond to BMI increments across specific gestational windows: **(a)** 10–13 weeks, **(b)** 13–20 weeks, **(c)** 20–24 weeks, **(d)** 24–28 weeks, **(e)** 28–32 weeks, **(f)** 32–36 weeks, and **(g)** 36 weeks to delivery.

The correlations between BMI measured at the first antenatal visit (10–13 weeks of gestation), stage-specific BMI increments, and neonatal birth weight are illustrated in **Figures 2 (A–G)**.

From the overall trend, a distinct stage-specific pattern was observed, characterized by a “strong–weak–strengthened” trend across gestation. Specifically, early-pregnancy BMI exhibited a clear positive correlation with neonatal birth weight, as reflected by an upward trend in the scatter distribution; in contrast, correlations during mid-pregnancy were weak and dispersed; while in late pregnancy, the association re-emerged and gradually strengthened, indicating a progressive contribution of maternal weight gain to fetal growth in the later stages of gestation.

### 3.5 Predictive value of early-pregnancy BMI and late-pregnancy BMI increments for macrosomia

To further assess the predictive effectiveness of BMI-related indicators across different gestational periods for macrosomia, ROC curve analysis was conducted.

**Figure 3:**
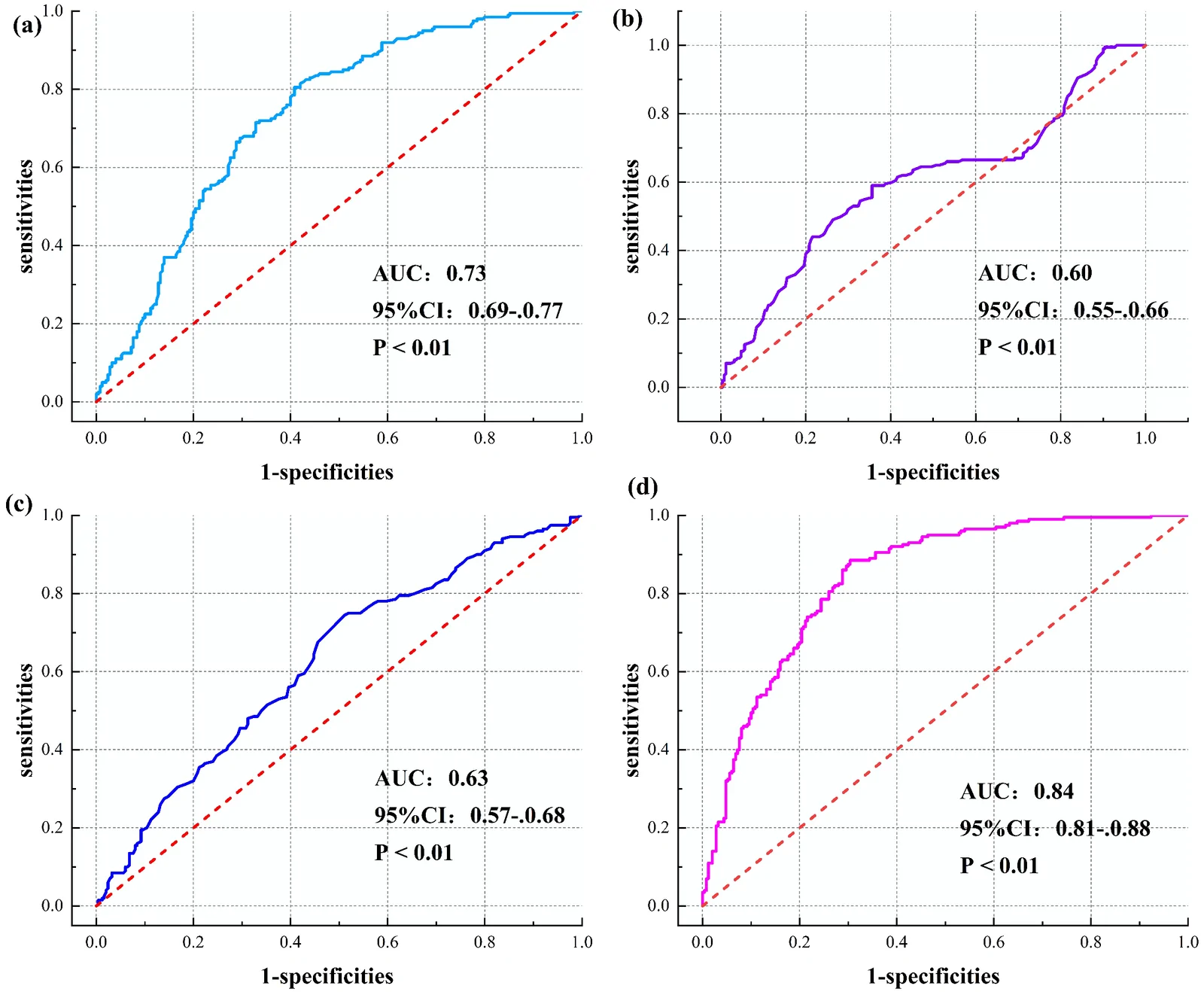
ROC curves for predicting fetal macrosomia based on BMI indicators: **(a)** BMI at the first prenatal visit (10–13 weeks of gestation); **(b)** BMI increment from 32 to 36 weeks of gestation; **(c)** BMI increment from 36 weeks of gestation to delivery; and **(d)** combined three-factor model.

The results indicated that BMI at the initial antenatal visit (10–13 weeks of gestation) exhibited favorable predictive performance for macrosomia, with an AUC of 0.73 (95% CI: 0.69–0.77, P < 0.01) (**Figure 3A**), reflecting a moderate discriminative ability. Among the late-pregnancy indicators, BMI increment from 36 weeks to delivery demonstrated the second-highest predictive value (AUC = 0.63, 95% CI: 0.57–0.68, P < 0.01) (**Figure 3C**), whereas BMI increment during 32–36 weeks showed comparatively lower performance in terms of prediction (AUC = 0.60, 95% CI: 0.55–0.66, P < 0.01) (**Figure 3B**). Notably, when these three indicators were integrated to establish a combined predictive model, the overall performance was substantially enhanced, with an AUC of 0.84 (95% CI: 0.81–0.88, P < 0.01) (**Figure 3D**), indicating strong discriminative capacity. The combined model displayed a steeper ROC curve relative to individual indicators, suggesting that BMI-related variables at different gestational stages offer complementary predictive contributions across time.

## 4 Discussion

Fetal macrosomia has been demonstrated to be associated with a substantially increased risk of poor maternal and neonatal outcomes (11). From a maternal perspective, the incidence of cephalopelvic disproportion and shoulder dystocia is markedly elevated, and inappropriate management of shoulder dystocia may result in severe vaginal and perineal lacerations or even uterine rupture (12). In addition, excessive uterine distension may lead to uterine atony and prolonged labor, thereby increasing the risk of severe postpartum hemorrhage (13). For the neonate, macrosomia often necessitates operative delivery and is associated with birth injuries such as intracranial hemorrhage, clavicular fracture, and brachial plexus injury, which may lead to serious morbidity or even mortality (14). Appropriate gestational weight gain (GWG) is a fundamental requirement for normal fetal growth. It is also pivotal in minimizing pregnancy-related complications while improving both maternal and offspring outcomes in the short and long term (15). Increasing evidence has demonstrated that inappropriate gestational weight gain exerts a substantial impact on pregnancy outcomes (16). Excessive increases in maternal BMI during pregnancy may contribute to vascular endothelial dysfunction and impaired uteroplacental perfusion, thereby affecting intrauterine fetal growth (17). Moreover, excessive fetal growth may lead to abnormal fat accumulation, ultimately resulting in macrosomia (18). A substantial retrospective cohort study undertaken in the United States, encompassing births from 1971 to 2017, documented an overall macrosomia incidence of 9.6% (19). It has been demonstrated by preceding studies that macrosomia is a multifactorial condition arising from the joint influence of pre-pregnancy baseline status and gestational weight regulation. Of these factors, excessive gestational weight gain is particularly salient as a contributing factor (20). Therefore, optimizing nutritional intake and promoting appropriate physical activity throughout pregnancy are essential strategies for reducing the risk of macrosomia. In the present study, we retrospectively examined pregnant women with uncomplicated singleton term pregnancies and systematically analyzed the associations between pre-pregnancy BMI, stage-specific BMI changes, and the macrosomia occurrence. The findings presented herein provide significant evidence to support the development of targeted gestational weight management strategies aimed at lowering macrosomia incidence and improving maternal and neonatal outcomes.

### 4.1 Association between pre-pregnancy BMI and macrosomia

As indicated by the findings of preceding studies, an elevated BMI prior to conception has been identified as a significant risk factor for the development of fetal macrosomia (21). A substantial body of research has emerged that suggests a positive correlation between pre-pregnancy BMI and neonatal birth weight (22), and that the presence of overweight and obesity prior to pregnancy can serve as independent predictors of macrosomia (23).

In the present study, after adjusting for relevant confounding variables, including maternal age, gestational age, gravidity, and parity, and using the normal-weight group as the reference category (OR = 1.00), a significantly elevated risk of macrosomia emerged higher among women with pre-pregnancy overweight (OR = 2.63, P < 0.001) and further increased in those classified as obese (OR = 9.06, P < 0.001). The findings demonstrate a robust positive correlation between pre-pregnancy BMI and the risk of macrosomia, thereby confirming that overweight and obesity before pregnancy act as independent contributing factors. This finding aligns with the observations reported by Badr et al., who demonstrated that pre-pregnancy obesity significantly increases the risk of delivery complications related to macrosomia (24). In a similar vein, Mazzone et al. reported that a higher pre-pregnancy BMI is associated with a significantly increased probability of excessive fetal growth, which corresponds with the elevated incidence observed in the obese group in our study (25). Although pre-pregnancy BMI has been consistently shown to correlate positively with neonatal birth weight (26), neonatal birth weight is influenced by a range of interacting factors, and the biological mechanisms underlying the correlation between pre-pregnancy BMI and fetal overgrowth remain to be fully elucidated.

### 4.2 Association between gestational BMI increments and the risk of fetal macrosomia

As demonstrated in previous studies, gestational weight gain exceeding the recommended threshold of 9 kg (with an upper limit of 25 kg) has been shown to be associated with an approximate 20% increase in severe maternal morbidity (27, 28). However, many pregnant women remain insufficiently aware of the risks related to excessive gestational weight gain, often relying on family guidance or traditional beliefs rather than evidence-based medical recommendations (29). It is widely acknowledged that both elevated pre-pregnancy BMI and excessive weight gain during pregnancy have been consistently identified as significant factors associated with fetal macrosomia. It is therefore recommended that appropriate weight management throughout gestation is implemented in order to mitigate adverse pregnancy outcomes (30). In women who are overweight or diagnosed with gestational diabetes mellitus (GDM), combined dietary and exercise interventions have been reported to lower the risk of macrosomia by approximately 15% (31). A substantial body of research has corroborated the notion that excessive gestational weight gain is demonstrably associated with an augmented likelihood of macrosomia (32, 33). Nevertheless, existing research has predominantly focused on total gestational weight gain, with relatively limited attention to stage-specific weight gain patterns during pregnancy (34). This gap hinders the development of individualized and dynamic weight management strategies in clinical settings.

In the present study, it was demonstrated that BMI upon the first antenatal visit (10–13 weeks of gestation) exhibited a moderate positive association with neonatal birth weight (r = 0.386, R² = 0.149, 95% CI: 0.304–0.462, P < 0.001), thereby indicating that early-pregnancy BMI possesses predictive relevance for neonatal birth weight. The maternal nutritional status in the early stages of pregnancy exerts a sustained influence on fetal growth in later stages. At this stage, BMI reflects not only pre-pregnancy body composition but also the initial metabolic milieu, which plays a crucial role in shaping fetal growth trajectories.

By contrast, no significant associations were identified between BMI increments during mid-pregnancy and neonatal birth weight (all P > 0.05), suggesting that fetal growth during this period remains relatively stable and less responsive to short-term maternal weight variations. As indicated by previous studies, the presence of excess adipose tissue in women with elevated BMI has been demonstrated to result in the secretion of pro-inflammatory adipokines, including leptin, tumor necrosis factor-α (TNF-α), and interleukin-6 (IL-6). Concurrently, these studies have also observed a reduction in adiponectin levels (35). These alterations can directly interfere with trophoblast invasion and spiral artery remodeling during early pregnancy, thereby modifying placental hemodynamics and nutrient transport efficiency (36). Consequently, elevated BMI in early pregnancy may predispose the fetus to enhanced nutrient uptake through epigenetic regulation and placental adaptations.

Furthermore, the present study showed that BMI increments during late pregnancy were significantly associated with neonatal birth weight. Specifically, BMI increment at 32–36 weeks of gestation exhibited a modest yet statistically significant relationship with neonatal birth weight (r = 0.171, P < 0.001), and this association became more pronounced from 36 weeks to delivery (r = 0.200, P < 0.001). The findings indicate that accelerated weight gain in late pregnancy is associated with increased fetal fat deposition and accelerated growth, signifying a critical “amplification phase” in the development of macrosomia.

From a physiological perspective, excessive energy intake during late pregnancy leads to an increased nutrient availability, comprising free fatty acids, amino acids, and glucose, which are transported across the placenta to the fetus (37). This phenomenon, often described as “nutrient surplus”, stimulates fetal pancreatic β-cell activity, resulting in fetal hyperinsulinemia and activation of insulin-like growth factors (IGFs), ultimately promoting excessive adipose accumulation and increasing the risk of macrosomia.

These findings emphasize that late pregnancy represents the final and most critical window for preventing macrosomia. Even if weight control during early and mid-pregnancy is adequate, insufficient monitoring and intervention in late pregnancy may still result in excessive fetal growth. In accordance with the findings of preceding studies, a substantial increase in weight gain during the second and third trimesters of pregnancy, particularly when this continues into the late gestational period, has been demonstrated to result in a notable elevation in the risk of infants being born with a large-for-gestational-age (38, 39).

### 4.3 Predictive value of gestational BMI indicators for macrosomia and their clinical implications

Excessive maternal body weight before pregnancy, together with excessive weight gain during gestation, are associated with enhanced fetal growth and an increased risk of macrosomia. On one side, these factors are directly associated with excessive fetal growth (40); on the other, they are linked to a higher likelihood of maternal metabolic abnormalities, including impaired glucose tolerance, GDM, and hypertensive disorders of pregnancy, thereby increasing the probability of non-elective cesarean delivery (41). A substantial body of evidence has indicated that pre-pregnancy overweight and excessive gestational weight gain are strongly correlated with adverse pregnancy outcomes (42). Specifically, excessive increases in maternal BMI during pregnancy have been related to pathological obstetric conditions, macrosomia, and delivery complications (43). It has been established through previous studies that pre-pregnancy overweight, advancing gestational age, excessive gestational weight gain, and hypertriglyceridemia in combination with GDM are key risk determinants for macrosomia (44). It is therefore vital that effective regulation of pre-pregnancy BMI and gestational weight gain is implemented in order to reduce the risk of macrosomia, since higher baseline maternal weight and greater weight gain during pregnancy are associated with a higher probability of delivering macrosomic infants (45). Recently, there has been an increased focus on gestational weight management in the field of perinatal medicine. The Institute of Medicine (IOM) has developed guidelines recommending specific weight gain thresholds during pregnancy, categorized according to pre-pregnancy BMI levels. However, these recommendations do not provide a clear definition of the optimal weight gain trajectories throughout gestation, nor do they identify the specific gestational periods with the greatest impact on neonatal birth weight. Consequently, the relationship between stage-specific maternal weight changes and neonatal birth weight has become a key research focus, although findings remain inconsistent across studies (46).

In the present study, ROC curve analysis demonstrated that, among individual indicators, BMI upon the first antenatal visit (10–13 weeks of gestation) exhibited the highest predictive capacity for macrosomia (AUC = 0.73, 95% CI: 0.69–0.77), with BMI increments during the latter stages of pregnancy demonstrating a subsequent predictive capacity. It is noteworthy that the combined predictive model incorporating early-pregnancy BMI and late-pregnancy BMI increments exhibited considerably enhanced predictive efficacy, with an AUC of 0.84 (95% CI: 0.81–0.88). From a clinical perspective, BMI at 10–13 weeks primarily reflects baseline maternal metabolic status and pre-pregnancy nutritional reserves, serving as an indicator for early risk stratification. In contrast, BMI increments after 32 weeks more accurately represent maternal energy supply during the phase of rapid fetal growth and act as dynamic indicators of risk progression. The integration of these indicators enables simultaneous evaluation of early risk initiation and late-stage risk amplification, thereby substantially enhancing predictive accuracy. The findings of this study are consistent with those reported in a prospective study by Sun et al. (47), which demonstrated that a 1 kg/m² increase in BMI at 12–14 weeks of gestation was associated with a 12.3% increase in macrosomia risk (95% CI: 1.08–1.17). Other studies have similarly confirmed a significant positive association between gestational weight gain and neonatal birth weight, underscoring the critical importance of appropriate weight gain in fetal development (48, 49). The underlying mechanisms may be explained as follows. During pregnancy, hormonal changes and reduced physical activity may exacerbate excessive weight gain, further aggravating insulin resistance and leading to disturbances in maternal glucose metabolism (50). In a hyperglycemic maternal environment, glucose is transported across the placenta in large amounts, resulting in compensatory hyperplasia of fetal pancreatic β-cells and elevated insulin secretion. Fetal hyperinsulinemia has been associated with increased synthesis of proteins and lipids, thereby increasing the risk of macrosomia (51, 52). Furthermore, excessive accumulation of maternal adipose tissue has been demonstrated to stimulate immune cell activation, particularly macrophages, resulting in increased secretion of pro-inflammatory cytokines, principally tumor necrosis factor-α (TNF-α) and interleukin-6 (IL-6). These inflammatory factors may traverse the placental barrier and interfere with normal fetal metabolic regulation, thereby facilitating adipose tissue deposition and further elevating the risk of macrosomia (53). Importantly, the timing of intervention in gestational weight management plays a crucial role. Early intervention is associated with greater improvements in pregnancy outcomes. The first prenatal visit at 10–13 weeks represents a critical window for identifying high-risk individuals through BMI screening and initiating individualized weight management strategies, thereby preventing excessive gestational weight gain from the outset. Meanwhile, the period from 36 weeks to delivery constitutes the final window for weight control, during which appropriate management is associated with reduced late fetal fat accumulation, decreased the severity of macrosomia, and ultimately lowered the likelihood of complications such as shoulder dystocia, birth canal injury, and neonatal trauma.

Taken together, although single-time-point BMI indicators provide limited predictive value, the integration of early-pregnancy BMI and late-pregnancy weight gain information offers a more comprehensive reflection of the intrauterine growth environment. This combined approach represents a superior strategy for predicting macrosomia and provides an important basis for establishing stage-specific, dynamic weight management and risk assessment systems in clinical practice.

### 4.4 Impact of excluding patients with metabolic complications on study findings and model generalizability

In addition to maternal BMI-related indicators, a growing body of evidence suggests that maternal dysglycemia affects not only fetal growth but also embryogenesis and organ development.

Previous studies have shown that diabetes-related microvascular dysfunction is significantly associated with adverse pregnancy outcomes, indicating that maternal metabolic status may influence embryonic development through the impairment of the maternal–placental–fetal microvascular axis (54). Recent evidence suggests that HbA1c provides a more stable assessment of glycemic-related risks compared with single-point glucose measurements (55). Elevated HbA1c levels during pregnancy may therefore indicate persistent intrauterine hyperglycemic exposure, which is closely associated with fetal overgrowth. Consequently, in such populations, the relative contribution of BMI changes to fetal growth may be partially masked or amplified by the effects of metabolic disorders, thereby altering its effect size and predictive thresholds.

This study strictly excluded patients with GDM, hypertensive disorders of pregnancy, and other known metabolism-related complications. This approach aimed to minimize the significant interference of hyperglycemia, insulin resistance, and systemic metabolic disturbances on fetal growth, thereby allowing for a more precise evaluation of the independent association between dynamic maternal BMI changes and fetal overgrowth.

The predictive model developed in this study—integrating early-pregnancy BMI and late-pregnancy BMI increments—essentially reflects the dynamic relationship between maternal energy reserves and nutritional supply during the rapid phase of fetal growth. This biological pathway shares a certain degree of commonality across diverse obstetric populations. Therefore, the model may still retain some applicability in populations with GDM or other high-risk pregnancies. However, its predictive performance parameters (such as AUC and optimal cut-off values) may shift, necessitating further calibration and external validation in different cohorts.

### 4.5 Study limitations

Despite the findings of the present study, which elucidated the relationships between BMI indicators at various gestational stages and macrosomia risk, along with their clinical relevance, it is important to consider the study’s limitations (56). Firstly, this investigation was conducted as an analysis of a single centre, and the study population displayed a relatively high degree of regional uniformity, which may restrict the broader applicability of the findings. Secondly, although several significant confounding variables, including maternal age, gestational age, gravidity, and parity, were incorporated into the analysis, data on several key lifestyle-related variables—particularly maternal dietary patterns and physical activity levels during pregnancy—were not included. Existing evidence indicates that both dietary intake structure and daily physical activity levels not only directly influence gestational weight gain trajectories, but also indirectly affect fetal growth and development by modulating insulin sensitivity, lipid metabolism, and inflammatory status. Therefore, the absence of these variables may have introduced some degree of residual confounding bias, thereby affecting the precise estimation of the association between BMI changes and neonatal birth weight.

Consequently, future studies should adopt multicentre, large-scale prospective cohort designs, incorporating populations from diverse geographic regions and socioeconomic backgrounds (57). In addition, external validation of the present predictive model is essential. Before clinical implementation, the model should be further evaluated in independent multicentre prospective cohorts to assess its generalizability, calibration performance, and robustness across different populations: including stratified modeling and external validation in populations with GDM and other metabolic abnormalities, alongside the systematic incorporation of prenatal dietary intake assessments and physical activity monitoring. Only after such validation can the model be recommended for routine clinical application in obstetric practice.

## 5 Conclusions

In pregnant women with singleton term pregnancies without complications, the occurrence of fetal macrosomia is closely associated with pre-pregnancy BMI, early-pregnancy BMI (10–13 weeks of gestation), and BMI increments during late pregnancy (from 32 weeks to delivery). Among these indicators, BMI at the first prenatal visit (10–13 weeks) may serve as a key parameter for early risk identification.

These findings indicate that gestational weight control should prioritize two key periods, namely early pregnancy and late pregnancy. The adoption of individualized weight management approaches during these stages, together with appropriate monitoring and targeted interventions, has the potential to effectively lower the incidence of macrosomia while improving both maternal and neonatal outcomes.

Overall, integrating early-pregnancy BMI with late-pregnancy BMI increments provides a practical and effective approach for risk stratification and clinical decision-making, offering important guidance for optimizing perinatal care and improving pregnancy outcomes. However, before this model can be implemented in routine clinical practice, it requires further validation through large-scale, multicentre, prospective studies to confirm its external validity and clinical utility across diverse populations.

## Data Availability

The original contributions presented in the study are included in the article/supplementary material. Further inquiries can be directed to the corresponding author.
